# Acceptability of oral HIV self-testing among female sex workers in Gaborone, Botswana

**DOI:** 10.1371/journal.pone.0236052

**Published:** 2020-07-27

**Authors:** Emily Shava, Kutlo Manyake, Charlotte Mdluli, Kamogelo Maribe, Neo Monnapula, Bornapate Nkomo, Mosepele Mosepele, Sikhulile Moyo, Mompati Mmalane, Till Bärnighausen, Joseph Makhema, Laura M. Bogart, Shahin Lockman

**Affiliations:** 1 Botswana Harvard AIDS Institute Partnership, Gaborone, Botswana; 2 Harvard T.H. Chan School of Public Health, Boston, MA, United States of America; 3 Nkaikela Youth Group, Gaborone, Botswana; 4 Ministry of Health & Wellness, Gaborone, Botswana; 5 University of Botswana, Gaborone, Botswana; 6 University of Heidelberg, Heidelberg, Germany; 7 Wellcome Trust Africa Centre for Health and Population Studies, Durban, South Africa; 8 RAND Corporation, Santa Monica, CA, United States of America; 9 Brigham and Women's Hospital, Boston, MA, United States of America; University of Ghana College of Health Sciences, GHANA

## Abstract

**Background:**

HIV prevalence among female sex workers (FSW) in sub-Saharan Africa is much higher than in the general population. HIV self-testing (HIVST) may be useful for increasing testing rates in FSW.

**Methods:**

We conducted semi-structured in-depth interviews among FSW, nurses and lay counsellors providing services to FSWs in Botswana. We aimed to gain understanding of perceived acceptability, anticipated barriers, and preferred approaches to HIVST among FSW. Interviews were audio-recorded, transcribed and translated. Transcripts were reviewed and coded independently by two investigators; high inter-coder agreement was achieved (Kappa = 0.80).

**Results:**

We interviewed five care providers whose average age was 40 years (SD = 2,64, range = 37–43); three nurses and two counsellors. Thirty FSW were interviewed, with mean age 34 years (range = 20–52). Most (27; 90%) FSW expressed great interest in using HIVST kits. Facilitators of HIVST were: awareness of own risky sexual behaviours, desire to stay healthy, and perceived autonomy over one’s healthcare decisions. Perceived advantages of HIVST included convenience, privacy, and perception of decreased stigma. Identified barriers to HIVST included lack of knowledge about the HIVST kit, fear of testing due to anticipated stigma, mistrust of the test’s accuracy, doubt of self-competency to perform HIVST, and concerns about not linking to care. Assisting someone to test was noted as good for providing emotional support, but there were concerns about confidentiality breaches. Providers expressed concerns over low literacy among FSWs which could affect comprehension of testing instructions, and competency to perform testing and interpret results. Participants’ recommendations for implementation of HIVST included: ensuring wide dissemination of information on HIVST, engaging peers in information-sharing and education, making test kits accessible in FSW-friendly centres, and having clear instructions for linkage to healthcare and support.

**Conclusion:**

HIVST shows high acceptability among FSWs in Gaborone Botswana, with providers expressing some concerns. Implementation should be peer-driven with healthcare provider oversight.

## Introduction

Knowledge of one’s HIV status is central to effective HIV prevention and care. HIV self-testing may be useful for increasing the frequency of testing in persons at risk of acquiring HIV, and for screening individuals who may infrequently engage with routine care. Heterosexual transmission is the main mode of HIV transmission in sub-Saharan Africa, and sex work is a driver of the epidemic. [1–4]

Female sex workers (FSW) in sub-Saharan Africa have a very high burden of HIV, [5] with HIV prevalence estimated to be at least three times that of general population [6–10]. Recent years have seen increased research in this key population in various sub-Saharan African countries. [11–15] However, the recent knowledge base related to HIV in FSW is quite variable by country, and little is known about current HIV testing trends and preferences in this key population in Botswana, a country with one of the highest HIV prevalence rates in the world. [16, 17]

HIV testing is a significant barrier to HIV prevention and care in FSW [16, 18–20] and HIV self-testing could provide a means to improve HIV testing coverage among FSW. HIV self-testing has been approved as a screening test per WHO guidance. [21] It has been shown to be feasible and acceptable in the general population and in high risk populations in the region [22–25] [11, 24] [26, 27], but has not been well studied in the Botswana context. Only two published studies have focused on FSW in Botswana. [7, 28] One of the studies showed that, although most FSW had been tested for HIV at least once (and had HIV prevalence of ~60%), regular HIV testing was infrequent. [7] The other study was conducted amongst 17 FSW in a mine town in Botswana: two focus groups and five in-depth interviews were conducted, and participants expressed negative attitudes towards HIV self-testing due to lack of knowledge and confidence to perform the testing independently. [28] Establishing the acceptability of HIV self-testing among FSW population in the country is of great importance as self-testing has the potential to increase early detection of HIV in the FSW population and other high risk, hard to reach groups.

We aimed to assess the acceptability of, obstacles to, and preferred approaches to HIV self-testing in FSW; and to assess whether FSW would use and/or share HIV self-test kits with others in Gaborone, Botswana.

## Methods

We conducted semi-structured in-depth face to face interviews using interview guides, between February and March 2019 among 30 consenting HIV-negative FSWs and 5 health workers at non-governmental organizations (NGOs) that are involved in providing services to FSW in Gaborone, Botswana. The study was conducted collaboratively by the Botswana Harvard AIDS Institute Partnership and the Nkaikela Youth Group, an organization which provides health care and social support to FSW in the Gaborone area.

### Setting

This study was conducted in Gaborone, the capital city of Botswana. An estimated 2,000 FSW work in and around Gaborone. [29] Nkaikela Youth Group provides services to over three quarters of the FSW population around Gaborone. [29]

Three individuals, two of which worked at the Nkaikela Youth Group conducted the interviews These individuals all had extensive experience working with FSW and were trained in conducting in depth interviews. Interviews were conducted in a private room at the Nkaikela Youth Group premises or at the Botswana Harvard AIDS Institute Partnership clinical research site. Interviews were audio-recorded.

### Participants and recruitment

To be eligible for the study, participants needed to be 18 years-old or older and identify as a FSW. Specifically, they needed to confirm receipt of money or goods in exchange for sexual services within the past three months, and consciously define those activities as income-generating even if they did not consider sex work as their occupation. FSW participants were recruited from Gaborone and surrounding areas through peer FSW outreach workers affiliated with the Nkaikela Youth Group. Peer outreach workers were trained on the recruitment process and eligibility criteria for the study. Potential participants were approached; if they met the criteria and provided informed consent, they were enrolled into the study. A convenience sample of participants was selected with the aim of representing FSW of different age groups and from different sexual networks.

Eligibility criteria for care providers included being involved in health service provision (e.g., lay counsellor or nurse) for FSWs in Botswana for at least six months. Care providers were recruited from organisations working directly with FSW in Gaborone Botswana. A convenience sample of participants was selected for in-depth interviews with the aim of representing different organisations and age groups.

### Data collection and qualitative analysis

Interviews were conducted in the language preferred by the participant (Setswana or English, or a mix of both languages), and were audio recorded by trained research assistants. Recordings were transcribed in the language in which they were performed, and translated into English. Translations were verified by a different bilingual native Setswana speaker who was not involved in the transcription and translation. Participants’ real names or other personal identifiers were not used in the interviews, and the recordings were stored on a secure password-protected computer by participant study identification number, accessible only to research staff. Participant study identification numbers were used on all records.

The investigators developed interview guides for both FSW and care providers that contained questions about perceived barriers to and facilitators of the use of oral HIV self-testing. Study participants were shown the oral HIV self-test kit (OraQuick). FSW participants were also asked about the perceived acceptability of sharing oral HIV self-test kits. Sharing was defined as either assisting someone to test, or giving someone an oral HIV self-test kit to use on their own (distribution of self-test kit).

We used standard qualitative analysis methods to identify potential barriers to and facilitators of oral HIV self-testing and sharing of oral HIV self-test kits. Two investigators (who were not directly involved in collecting the data) separately read all transcripts to identify themes using the research objectives, looking for repetition across interviews and examples of processes, behaviours, and cultural assumptions. The investigators each independently developed initial listings of themes, then develop a codebook listing each theme accompanied by a detailed description, inclusion/exclusion criteria, and typical examples. The investigators each wrote independent summaries of themes. Results were reviewed to evaluate intercoder agreement between the coders, and consensus in the coding and interpretation of results was reached amongst the investigators.

The emerging codes were grouped under 39 subthemes. The subthemes were further grouped under the following six broader themes: barriers to oral HIV self-testing, facilitators of oral HIV self-testing, perceived advantages of oral HIV self-testing, desire to perform oral HIV self-test, sharing of test kits, and recommendations for and preferred approaches to oral HIV self-testing.

### Ethical statement

The “Ikitse Study “was approved by the following institutional review boards: the Botswana Ministry of Health and Wellness, Human Research Development Committee and the Office of Human Research Development Administration at the Harvard T.H. Chan School of Public Health. All participants taking part in the study provided written informed consent.

## Results

### Participant characteristics

Thirty-five participants took part in the in-depth interviews (30 FSW and 5 providers).

Among the 30 FSW participants, the mean age was 34 years-old (SD = 8; range = 20–52). Of the 5 providers interviewed, the average age was 40 years (SD = 2,64, range = 37–43); three were nurses (2 female, 1 male) and two were counsellors (2 female).

### Attitudes towards HIV self-testing (FSW)

The vast majority of FSW had a positive view of oral HIV self-test kits: nearly all (27/30) FSW expressed great interest in using oral HIV self-test kits. One participant said, *“…*.*I want it like a child yearning for breast milk*. *I don’t know how to explain but I would be happy to test myself*.*”*

Two FSW were undecided about oral HIV self-testing. One FSW said that she would definitely not use oral HIV self-test kits because she would not link to care if she had a reactive result.

### Perceived facilitators and advantages of and barriers to HIV self-testing (FSW)

FSW noted several facilitators of HIV self-testing (Table 1). Key facilitators of HIV self-testing expressed by FSW were being aware of one’s risky sexual behaviour (expressed by all 30 women) and a desire to stay healthy (by 22 women). Autonomy and convenience associated with self-testing were felt to be advantages of self-testing (by 16 and 30 women, respectively). Regarding convenience one participant said “..at hospitals nowadays their (HIV) test kits are for pregnant women, several times, about twice or thrice when I wanted to test they said their testers are for pregnant women. (HIV) Self-test kit I feel can be very helpful even for the public..”

**Table 1:** Perceived barriers and facilitators regarding HIV self-testing among 30 FSW and 5 healthcare providers.

Parent Theme	Sub-theme	FSW quote	Healthcare provider quote
**Facilitators of HIV Self-testing**	1. Self-perception of risky sexual behaviour	*Some of us have sex with men in exchange for cash*, *we don’t engage in sex for love; saying we are building families*, *we are concerned about money so when it’s like that you have to know your status*.	*I think it is the level of risk of their job*, *because they are at a higher risk compared to the general public*, *so at least it can motivate them to do the test because they are at risk*.
	2. To stay healthy for family/self	*Women get pregnant and they can protect their babies and their lives*.	*They [FSW] are actually the people who really want to access the health care services it’s just that there are some structural barriers that prevent them from accessing those health care services*.
**HIV self-testing—perceived advantages** versus testing at healthcare facility	1. Convenience	*This thing is like you will be at home; you will be testing yourself at home and then you’d know your status…*..*you don’t have to go to the clinic and queue or being told the nurse is not in*, *come back tomorrow or do this and that; getting on combis to get there you see*.	*They are going to use it because it’s very convenient*, *can you go queue the whole day there while you have something to use at home*?
	2. Autonomy	*It is good because you satisfy yourself by doing the HIV self-testing than being tested by another person*.	*Some people prefer self-testing…because they have that fear of being told the test results by someone else*.
	3. Decreases stigma	*It is better because some people feel ashamed to go and queue at the clinic or if they do not have time or they just feel lazy to do it*.	*I think they can like it (HIV self-testing) because most of the times they feel when they go to clinics*, *they are judged*, *being judged is because whenever people see them*, *they look at them as people who want sex*, *and they look down upon them*, *so*, *sometimes when you have self-tested at home*, *it is better when you go when you have already accepted yourself*.
	4. Private/confidential	*There can be great benefits since someone will have a chance to test in their own privacy and space without any distractions*	*People still don’t understand a lot about confidentiality in health facilities*, *so they just think that when you go for a test…*.*(health care personnel) will go around telling people about my results like telling other people my status*.
	5. Easy to use/not much training needed	*Because it is easy…*.*as you do it on yourself while at home and wait for you result*.	N/A
	6. Motivation for more frequent testing	*It’s not like I want people to know what I do and where*. *…*..*So me constantly going to the clinic every month*. *The nurse could say to me you were just here testing yourself*, *are you here today to test yourself once again*. *So if I do it at home I feel like it has somehow protected me*.	*They can test regularly*, *if they have tested and they want to test again and when they think about the queue at the clinic then it will be something else*, *so they will be able to monitor themselves*
**Barriers to HIV Self-Testing**	1. Fear of testing/negative emotions/doubt of accuracy of test	*Fear*, *people would be scared*, *they will be afraid of how it will be if they turn out to be positive*. *A young person like myself*, *they think of those things*. *Fear is an obstacle*.	*Maybe the challenge will be*, *where are we going to store the test kits*, *where do they get them*, *there is a way they are supposed to be stored or kept under certain temperatures or specific places*, *so that they give valid results*, *so the challenge will be if someone is to test themselves where will they be getting them*, *do we trust that they will give correct results*.
	2. Doubt of competency in performing the test	*I will fail to go to the clinic to go and confirm my positive results*. *I will just sit home and dismiss it by saying; well*, *that thing is not accurate*, *after all*, *I am the one who did the test*.	*FSW …some are illiterate some semi-literate the issue goes back to reading*, *people don't read …*.*and you don't understand what exactly [you] are supposed to be doing then it becomes a problem*.
	3. Lack of linkage to care	*I can end up cheating myself*. *I may find myself “like that” and then cheat myself by not going to tell them at the clinic*, *so that I can get treatment*.	*(Linkage to care) is difficult…the problem is*, *they are forever in a hurry*, *so*, *it would be difficult for them to bring themselves*, *so*, *I don’t know how else we can do it*.
	4. Negative consequences due to Lack of pre and post-test counselling/ emotional support	*If it is to be given to a person*, *there should have been provided with counselling before giving her the kit like briefly tell her about those outcomes they should expect*, *you know there have been instances where you hear someone has killed themselves without any suicide note*.	*The only concern I have is it’s not everyone who will be ready to accept the test results*, *if they are positive I really have fear of how they are going to accept them…wouldn’t they end up having suicidal thoughts when there is no one there*, *you will never know ones reaction afterwards*.
	5. Lack of motivation to get tested	*You can say to a friend “I have tested earlier*, *maybe you should go and test” and they will say “nowadays HIV does not kill*, *only cancer kills”*.	*Listening to you mentioning the 20 to 40 minutes period that you need to wait for results …would key populations especially FSW be motivated to test…*
	6. Lack/limited knowledge of HIV Self-test kits use, storage	*You see this self-test kit*, *I don’t think majority of people know about it*.	*These women would like it but my challenge is they are going to use it but at the wrong time because I have noticed that most of the time when they are exposed to risk*, *the next day they would go for testing*, *so I don’t know how sensitive this kit is to an extent they when a person got exposed the day before can it can detect the virus but if it’s similar to the three months one*, *three months window period*, *they are going to end up abusing them because they test a day immediately after being exposed*.
	7. Fear of testing/negative emotions/doubt of accuracy of test	*Like now*, *I would want to give it to someone and they may say a lot of things*, *like you want to infect them with HIV*, *many people including me*, *is the first time I see this kit*, *so*, *a person will be like*, *this one tested inside the mouth*, *is news to me*, *most of us don’t know it*, *we only know the blood one*.	*Maybe the challenge will be*, *where are we going to store the test kits*, *where do they get them*, *there is a way they are supposed to be stored or kept under certain temperatures or specific places*, *so that they give valid results*, *so the challenge will be if someone is to test themselves where will they be getting them*, *do we trust that they will give correct results*.

FSW expressed a general interest in sharing test kits with others (such as with a friend or client). In one participant’s words “… *I can take my peers and make them queue and say*, *“pals*, *inside” (for HIV testing)*.*”* They preferred the idea of giving someone a test kit for their own use (distribution), rather than assisting someone else to HIV self-test. Assisting someone to test was noted to be good for providing emotional support but raised confidentiality concerns. All the FSW had no prior knowledge on HIV self-testing, and some of them expressed that limited knowledge can be a barrier to HIV self-testing. Fear of testing and lack of linkage to care were some of the barriers to HIV self-testing highlighted by the women.

### Health care providers

Providers cited similar perceived facilitators and advantages of HIV self-testing as FSW: risky sexual behaviour (5/5), convenience (4/5), and decreased stigma (3/5).

Providers expressed concerns regarding possible low literacy of FSW, which could affect the reading and comprehension of testing instructions and hence competency to properly perform the test and interpret the results:

“*They don't take time to read and understand*. *What I’m drinking doesn't have any side effects*? *How is it going to affect me*, *you know stuff like that*. *So I'm trying to imagine if someone would take that time and actually read and adhere to the instructions because most of the female sex workers …some are illiterate some semi-literate*. *So if the package comes in English*, *that means they will either need somebody to translate for them or try to summarize and break down the message for them and that on its own kind of clouds the environment*, *the convenience and private space*.*”*

Providers also expressed concern that patients who test positive may despair and/or not present for care. Training on how to store and use the HIV test kit and when to self-test were noted to be key. Providers also noted potential challenges of implementing assisted HIV self-testing of others, including the need for seeking permission and upholding confidentiality.

### Recommendations for implementing HIV self-testing (FSW and healthcare providers)

Table 2 summarizes participants’ recommendations for implementation of HIV-self testing. Both FSW and their care providers recommended that prior to implementation of oral HIV-self testing, there should be wide dissemination of information on HIV self-testing in general, e.g. through the media (TV, radio, billboards and fliers). In a participant’s words: *“Like now you can bring your caravan in an open space in Old Naledi and make a public announcement and tell them you are teaching about such and such tester*, *people would come and want to see the tester being talked about*, *how it is*, *how important it is*.*”* This information should include accuracy of the test, storage of the test kit, how and when to perform the test, how to dispose of the used test kits, and steps to take after obtaining test results. FSW stressed the importance of also having peers provide this information and education, with guidance from healthcare providers; one FSW suggested, *“Maybe you could pitch a tent not too far from the clinic*.*”* Both FSW and healthcare providers emphasized that it will be important to ensure that key populations have access to HIV self-test kits by making them available at centres or organisations that provide care and support to these populations.

**Table 2:** Participant recommendations for implementation of HIV-self testing.

Interview question/Emerging theme	FSW participants	Health Care Provider participants
Who can train FSW on HIV self-testing?	*Women can be trained by*, *especially the female sex workers*, *they can be trained by other sex workers who work here in Nkaikela for example*. *Because in our community*, *no one can come from outside to help you*, *they cannot*. *Another sex worker can help you*. *This is because the person from outside may say*. *Can I help a person who acquires diseases deliberately*?*”*	. *I think it should be someone who has been trained on HIV*, *not necessarily a health care provider or not necessarily someone with a clinical background*, *somebody who is knowledgeable about HIV acquisition like we have been discussing but anybody can train sex workers in self testing*.
What information/education do FSW require to use oral HIV self-test kit	*So we need to be taught how this one is done and how often it should be done…*. *Yes*. *And testing*, *when do we do it*? *Yes*.	. *They may need to be taught …for a person to able to accept something*, *just from the beginning*, *you should tell them the purpose*, *why the need*, *so they have been taught why and understanding they will accept this kit*, *and then be taught on how to use it*, *and then understand the benefits because if you don’t understand the benefits you will not be eager or have that desire to use it*.
How should Education on HIV Self Testing be delivered?	*Information can be spread by youth voluntary organizations/groups like those of Nkaikela*, *to approach a person*, *before you talk to him/her*, *they want to know who you are*, *why you are talking to them about HIV*, *why you want to give them self-testing*, *they would want to know…that’s why I say this self-kit can be given to women by this other organizations as it is known they go into homes and clinics*, *people should be educated about it so that they know about it and talk to people about it including those who are difficult to test*, *its difficult but also not difficult*, *I believe if you sit down with a person*, *they can understand the importance of testing for HIV*.	*Information should be disseminated through the media*, *radio*, *social media and sessions whereby female sex workers are educated*, *even education the general population*, *there’s going to be a need to educate the health care providers then they can spread it across clinic through morning talks*, *yes it can be done sharing it with people in that way*.
Distribution of Test kits	*It would be ideal for them to pick them up from protected/safe spaces like Nkaikela*.	*I have realized they don’t have a problem coming to a place like this NGO*, *they just come to access services*, *so*, *I can recommend it*. *When you talk about the clinic they think of waiting it a line that goes beyond the gate*, *so*, *it will be difficult for them…so places like NGOs and social services organizations if they had a place they can issue them*, *these are places that can help*.
Sharing of test kits	*To test other people*, *yes*, *I would do it…would test other sex-workers like me*. *As we meet up in the afternoons I would say to them “Friends*, *there is a thing that you can test yourself with without going to the hospital”…They are the people… the sex-workers*.	*I don’t have an objection against that*, *as much as someone has been given enough information*, *how to take it and how to interpret the results*, *then there is no problem at all*.
Linkage to care	*If it is me testing the other person*. *If I see that maybe the person is “like that”*, *I will talk to them and encourage them to go the clinic or to the hospital so that they can receive treatment and assistance*.	*Let’s have a telephone line*, *because they test at home they call this line or we can provide instructions on the test kit on how to link to care and be able to contact whoever by text or any means of communication to be able to communicate with whoever will be coordinating and then get linked (to care)*

They also highlighted the need to have clear instructions and messages on linkage to health care and support after HIV self-testing.

## Discussion

In this qualitative study, we elicited perceived barriers to, facilitators of, and advantages of oral HIV-self testing among FSW and their healthcare providers in Gaborone, Botswana. Oral HIV self-testing was highly acceptable to FSW and generally acceptable to their care providers. Women expressed that self-testing provides a convenient, private opportunity to test for HIV that is under their control, potentially facilitating more frequent HIV testing. This has also been shown through other studies conducted in a similar population. [30–33] Providers’ concerns included their perception of low literacy among the FSW, which was thought to affect the self-testing and resulting processes. This could explain why the acceptability of HIV self-testing by the providers was not overwhelming.

FSW in Botswana encounter various structural barriers in accessing much-needed HIV testing services. [34, 35] In the present study, HIV testing by providers in health facilities was noted to pose various barriers for this population, including duration of time spent in the clinic because of long queues, and perception of external stigma. These findings are consistent with prior literature. [32, 33, 36] Through this study we discerned a perception that some barriers, such as stigma and inconvenience, may be addressed by availability of HIV self-test kits. This is consistent with literature from other countries in the region. [12]

Major barriers to HIV self-testing included lack of knowledge about the oral HIV self-test kit itself. None of the FSW interviewed were aware of the existence of oral HIV self-testing, with all interviewed FSW not having previously seen an oral HIV self-test kit. This is unlike other FSW and high risk groups in the region. [11, 30, 37–39] This lack of knowledge about the test-kit and the associated anxiety can clearly be addressed by the recommendations made by the FSW themselves in this study, namely to ensure wide (and clear) dissemination of information. Additionally there may be need for implementors to lay a good foundation for the introduction of the self-test kits through awareness campaigns which may in turn affect uptake.

Despite concerns regarding linkage to care following a positive HIV self-test and lack of pre- and post-test counselling, FSW clearly expressed a preference for HIV-self testing in comparison to facility-based HIV testing. Testing in the presence of a trusted person or assisted HIV self-testing were noted as ways to ensure emotional support and enhance subsequent linkage to care for persons testing positive on the self-test. Linkage to care is clearly a key aspect in the successful management of FSW and further research in this area may be beneficial.

In this study, all FSW were aware of their risky sexual behaviour, the potential of becoming HIV infected, and the need to frequently test for HIV. This is also consistent with studies performed in the sub-Saharan African region. [28, 39, 40] Our study was not designed to assess the impact of this knowledge of risk on the frequency of HIV testing.

Both FSW and healthcare providers noted the importance of FSW peer involvement for any strategies in the implementation of HIV self-testing to be successful among FSW in Botswana. This recommendation could be as a result of general mistrust and/or as a result of the perceived stigma and discrimination against FSW from authorities. [41, 42] Addressing this recommendation could potentially improve social integration of the FSW communities which might in turn have a bearing on other health seeking behaviours.

The study enrolled women 18 years and older in and around Gaborone, Botswana via a convenience sample. Limitations of the study may include lack of generalisability of the study findings to adolescent FSW or to FSW in other parts of the country or the region. The FSW enrolled in the study were somewhat engaged in regular HIV testing, since they were linked to a support organization. Hence, our study population may largely reflect FSW who are not averse to HIV-testing in general. In addition, we interviewed five healthcare providers, mostly female. This small sample may be insufficient for understanding the range of healthcare provider perceptions.

## Conclusion

Oral HIV self-testing is a highly acceptable method of HIV testing among female sex workers. Future implementation of oral HIV self-testing among FSW should address the identified structural barriers in the healthcare system and associated knowledge gaps among FSW. Thus, implementation of this testing method should be peer-driven with oversight and guidance from healthcare professionals.

## Supporting information

S1 File (ZIP)
